# Xylo‐oligosaccharides improve functional constipation by targeted enrichment of *Bifidobacterium*

**DOI:** 10.1002/fsn3.3827

**Published:** 2023-11-27

**Authors:** Wanya Yi, Qinyue Wang, Yuzheng Xue, Hong Cao, Ruijuan Zhuang, Dan Li, Jiai Yan, Ju Yang, Yanping Xia, Feng Zhang

**Affiliations:** ^1^ Department of Nutrition Affiliated Hospital of Jiangnan University Wuxi China; ^2^ Wuxi School of Medicine Jiangnan University Wuxi China; ^3^ Functional Food Clinical Evaluation Center Affiliated Hospital of Jiangnan University Wuxi China; ^4^ Department of Gastroenterology Affiliated Hospital of Jiangnan University Wuxi China; ^5^ Department of Endocrinology Affiliated Hospital of Jiangnan University Wuxi China; ^6^ Department of Geriatrics Affiliated Hospital of Jiangnan University Wuxi China; ^7^ Yixing Institute of Food and Biotechnology Co., Ltd Yixing, Wuxi China

**Keywords:** *Bifidobacterium*, dietary care, functional constipation, gut microbiota, xylo‐oligosaccharides

## Abstract

Functional constipation (FC) has a negative impact on patients' quality of life. We hypothesized that dietary supplementation with xylo‐oligosaccharides (XOS) or fructo‐oligosaccharides (FOS) would improve constipation symptoms by influencing the gut microbiota. A randomized double‐blind controlled trial was conducted in FC patients. Patients were randomly divided into 6 groups and given a dietary supplement containing XOS at doses of 3, 5, or 10 g/day, FOS at doses of 10 and 20 g/day, or placebo at 5 g/day for one month. We compared improvements in gastrointestinal function after the intervention using the Bristol Stool Form Scale (BSFS), Cleveland Clinic Constipation Score (CCCS), and Quality of Life Scale for Patients with Constipation (PAC‐QoL). 16S rRNA sequencing was used to assess changes in the structure of the gut microbiota. Changes in individual bacteria had significant effects in reducing gastrointestinal symptoms during the intervention, even though the flora structure remained unchanged from baseline. Compared to FOS, XOS enriched *Bifidobacterium* at a lower dose, and patients receiving XOS supplementation showed significant improvements in constipation symptoms without side effects such as diarrhea and flatulence.

## Graphical abstract

We hypothesized that dietary supplementation with xylo‐oligosaccharides (XOS) or fructo‐oligosaccharides (FOS) would improve functional constipation (FC) symptoms by influencing the gut microbiota. FC patients were randomly divided into six groups and given a dietary supplement containing 3, 5, 10 g/day of XOS, 10 and 20 g/day of FOS, and 5 g/day of placebo for one month. We found that XOS dietary care could target enrich *Bifidobacterium* and improve constipation symptoms at lower dosages than FOS dietary care.

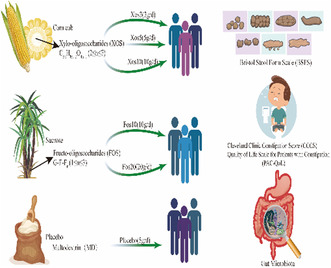

## 1 INTRODUCTION

Functional constipation (FC) is defined as constipation without organic etiology (Sperber et al., 2021), and it is a common disorder in children and adults (Wallace et al., 2022). The prevalence has been found to be 15.3% in studies that define FC according to Rome I criteria, 11.2% in studies using Rome II, 11.4% in studies using Rome III, and 10.1% in studies using Rome IV criteria (Barberio et al., 2021). FC has a negative impact on patients' quality of life and causes a significant financial burden (Abdullah et al., 2015; Long et al., 2023). Approximately 50% of patients with constipation are not completely satisfied with standard treatment (Johanson & Kralstein, 2007) due to lack of efficacy or safety concerns. All of these factors have prompted interest in pursuing alternative treatment strategies.

According to the Food and Agriculture Organization (FAO) of the United Nations, prebiotics are indigestible or low‐digestible food ingredients that can promote the growth of probiotics and improve human health through nutrient enrichment, and modulation of the gut microbiota and the host immune system (Yadav et al., 2022). In particular, prebiotic supplements that support the activity of *Bifidobacterium*, which is one of the most common probiotics, have shown beneficial physiological effects, including anti‐inflammation and anti‐depression activities, regulation of the host immune system, and maintaining of the intestinal microbial balance (Chen et al., 2021).

Typical prebiotic compounds include fructo‐oligosaccharides (FOS), xylo‐oligosaccharides (XOS), galacto‐oligosaccharides (GOS), and inulin, etc. FOS is a short polymerized soluble dietary fiber containing 3–10 fructan groups (Liu et al., 2022). Consumption of FOS has been shown to reduce gastrointestinal transit time and increase fecal weight (Meksawan et al., 2016). In a study of adult subjects, Bouhnik et al. (2006) found a significant correlation between the ingested dose of FOS and fecal *Bifidobacterium*. This bifidogenic effect appeared with a dose of FOS of 2.5 g/day, and a dose–response relationship was observed at doses from 2.5 to 10 g/day. Similarly, a meta‐analysis reported by Dou et al. (2022) found that FOS supplementation could increase the number of fecal *Bifidobacterium* while higher doses (7.5 g–15 g/day) and longer treatment durations (>4 weeks) showed more distinct effects and was well tolerated.

Another non‐digestible oligosaccharide, XOS, is an emergent prebiotic (Palaniappan et al., 2021) that is derived from plants and has been used in the prevention and treatment of several metabolic diseases. Like FOS, treatment with XOS has also been found to increase intestinal *Bifidobacterium* (Tong et al., 2022). The fecal water content was maintained within a normal range during the treatment period, and the frequency of stools increased by 4.2 g XOS daily intake (Tateyama et al., 2005). The effects of XOS on gastrointestinal parameters have been evaluated at multiple doses in several studies (Turck et al., 2018). XOS has been shown to be beneficial for bowel function, and the most tolerant administration dose for adult subjects was 12 g/day (Xiao et al., 2012).

Risk factors of FC, such as age, diet, lifestyle habits, and stress, can impact the gut microbiota (Vriesman et al., 2020). therefore, we hypothesized that prebiotic treatments could influence FC by modulating changes to the gut microbiota. Prebiotic agents have been used in other studies in this way. For example, Chu et al. (2019) found that UG1601, a combination of inulin, lactitol, and aloe vera gel, was effective in the treatment of patients with mild chronic FC (Chu et al., 2019). Similarly, the prebiotic activity of FOS has been reported to improve constipation symptoms in animal studies (Wang et al., 2017) and in human clinical trials (Reimer et al., 2020). On the other hand, while XOS exhibits a low digestibility in humans and may thus be effective at very low doses, this supplement has received little attention in FC‐related studies.

Each prebiotic has different effects on specific gut microbes, and exploring the effects of different prebiotics or mixtures of prebiotics can provide insight into ways to develop a more diverse and resilient microbiome. Therefore, the purpose of this study was to evaluate the impact of supplements containing FOS or XOS on the gut microbiota and FC symptoms.

## 2 MATERIALS AND METHODS

### 2.1 Clinical experimental design

This double‐blind, randomized, placebo‐controlled 1‐month clinical trial was conducted to compare the efficacy of dietary care patterns containing FOS and XOS in FC patients. Care‐givers led this trial and collaborate with nutrition department.

Patients were randomly assigned to six groups (Table 1). Randomization was performed with a simple sealed envelope method. A sealed, opaque, sequentially numbered envelope containing the treatment allocation was handed to an eligible participant. The nurse then entered the participant into the trial by opening the sealed envelope.

**TABLE 1:** Experimental design, g/day.

Group	Intervention
XOS3 group	Intake of a dietary supplement containing 3 g/day XOS
XOS5 group	Intake of a dietary supplement containing 5 g/day XOS
XOS10 group	Intake of a dietary supplement containing 10 g/day XOS
FOS10 group	Intake of a dietary supplement containing 10 g/day FOS
FOS20 group	Intake of a dietary supplement containing 20 g/day FOS
Placebo group	Intake of a dietary supplement containing 5 g/day placebo

*Note*: XOS3: supplementation with xylo‐oligosaccharides (XOS), 3 g/day, XOS5: supplementation with XOS, 5 g/day. XOS10: supplementation with XOS, 10 g/day, FOS10: supplementation with fructo‐oligosaccharides (FOS), 10 g/day, FOS20: supplementation with FOS, 20 g/day, Placebo: supplementation with placebo 5 g/day.

XOS, FOS, and Placebo are provided by Henan Heagreen Biotechnology Co, Ltd (Henan, China). The component of the placebo is maltodextrin. XOS source corn cob, XOS ≥ 95%, 5% xylose, arabinose; FOS source sucrose, FOS ≥ 95%, 5% glucose. During the experimental period, the subjects maintained their regular diet, and their daily food intake was not restricted. Weekly followed up visits by nurses. All available clinical cases with evaluable biospecimens during the period of data generation of this observational/correlative study were used.

Approval of the Human Subjects Committee of the Affiliated Hospital of Jiangnan University was obtained before the beginning of the study (Number: IEC201803001) and was registered at the Chinese Clinical Trial Registry (ChiCTR1800015888). Informed consent was obtained from all subjects who participated in the study.

### 2.2 Subjects

Patients aged 18–75 years with a confirmed diagnosis of FC according to the Rome IV criteria were enrolled at Affiliated Hospital of Jiangnan University in China. Patients were excluded if they were pregnant or breastfeeding; diabetic; taking antibiotics; taking medications that affect bowel motility, such as calcium antagonists, nitrates, antimuscarinic agents, high doses of stimulant laxatives (three times per week) within the past 5 years; abdominal and/or bowel surgery, appendectomy, cholecystectomy, pelvic floor dysfunctions, etc; unwilling to sign the informed consent form; unable to attend the study visits; had an allergy to any of the product ingredients; or whose health status precluded participation. The use of other laxatives, probiotics, fermented dairy products, and yogurt were not allowed during the study period, and a glycerin suppository was used only when there was no defecation for more than 3 days.

In order to calculate the sample size, we conducted a pre‐experiment. 30 patients with FC were assigned to six groups, with 5 persons per group. For sample size calculation, PASS 11 (NCSS, LLC. Kaysville, Utah, USA) was used. One‐way analysis of variance were used to compare the effects between different groups after intervention with Cleveland Clinic Constipation Score (CCCS) (Table S2). CCCS were 9.00 ± 3.81 in Group XOS3, 5.80 ± 2.49 in Group XOS5, 7.00 ± 2.74 in Group XOS10, 8.40 ± 1.67 in Group FOS10, 7.60 ± 1.52 in Group FOS10, 11.0 ± 4.00 in Group Placebo. With 80% statistical power and a two‐sided significance level (α) of 5% and consideration of 10% disengagement rate, we aimed to recruit 96 volunteers to complete the study.

### 2.3 Questionnaire design

Defecation and gastrointestinal function were evaluated using the Bristol Stool Form Scale (BSFS), Cleveland Clinic Constipation Score (CCCS), and Quality of Life Scale for Patients with Constipation (PAC‐QoL). During the research, care givers used Food Frequency Questionnaires (FFQ) to record patients' diets. Simultaneously, care givers monitored patients' gastrointestinal symptoms, appetite, and psychological status. The subjects were visited on time to evaluate the intervention efficacy.

The BSFS, which combines a picture with standardized descriptors of stool form, is a widely used, reliable, and validated patient assessment of stool consistency that can be used to approximate the presence of FC. The BSFS classifies the faces into one of seven stool types ranging from type 1 to type 7. Types 1 and 2 are considered to be abnormally hard stools (and in conjunction with other constipation‐related symptoms) while Types 6 and 7 are considered abnormally loose/liquid stools (alone with other diarrhea‐related symptoms). Type 3, 4, and 5 are generally considered to be “normal” stool form. Grading of constipation severity was performed using the CCCS which contains 8 items related to constipation (frequency of bowel movements, abdominal pain, type of assistance, etc.). CCCS gives a validated, incremental score ranging from 0, equating to no symptoms, to a maximum of 30, equating to severe symptoms (Agachan et al., 1996). The PAC‐QoL is a 28‐item scale designed specifically to assess the quality of life of patients with FC. It includes an overall scale and four subscales: worries and concerns (11 items), physical discomfort (4 items), psychological discomfort (8 items), and satisfaction (5 items) (Riezzo et al., 2019). Each item is scored on a 5‐point scale, with a higher score indicating the severity of the disease. The FFQ, based on the Dietary Guidelines for Chinese Residents, which query frequency and portion size of food items consumed during a defined period such as the past month. Usual dietary intake was assessed using a validated semiquantitative FFQ that listed 138 food items regularly ingested by Chinese (Shu et al., 2017). Each food item had nine options for frequency that ranged from “never” to “3 times/day” and three options for portion size (Li et al., 2019). Constipation‐related questionnaires were completed once before and after the intervention. FFQ completed once after the intervention.

### 2.4 Feces collection

Feces were collected in plastic containers that had been rendered anaerobic. On day 0 and day 30, fecal specimens from each patient were collected in the morning and wrapped in ice packs, until they were transferred to the −80°C refrigerator within 1 h. There were no intentional freeze–thaw cycles and all samples were measured together.

### 2.5 DNA extraction and 16S rRNA amplicon

The DNA of the fecal samples was extracted using a FastDNA Spin Kit for Feces (MP Biomedicals, LLC, Irvine, CA). 1% agarose gel electrophoresis was used to check the DNA integrity, and NanoDrop 2000 UV–vis spectrophotometer (Thermo Scientific, United States) was used to detect the DNA concentration and purity.

The hypervariable region V3‐V4 of the microbial 16S rRNA gene was amplified via an ABI GeneAmp® 9700 polymerase chain reaction (PCR) thermocycler (ABI, CA, United States) using the following primer pair: 341F, 5′‐ACTCCTACGGGAGGCAGCAG‐3′, and 806R, 5′‐GGACTACHVGGGTWTCTAAT‐3′. The protocol of PCR thermocycler was as follows: 3 min at 95°C followed by 29 cycles of 30 s at 95°C, 30 s at 55°C and 45 s at 72°C, and a final 5 min at 72°C. PCR reactions were performed in three replicates. Subsequently, the mixture PCR products were purified using the Qiagen Gel Extraction Kit (Germantown, MD, USA). Sequencing libraries were prepared by pooling all the samples using the TruSeq® DNA PCR‐Free Sample Preparation Kit (San Diego, CA, USA). And the library quality was measured using Agilent 2100 Bioanalyzer. Finally, the library was sequenced on an Illumina HiSeq 2500 platform and 250 bp paired‐end reads were generated (Liu et al., 2021) according to the standard protocols by HonSunBio Technology Co. Ltd (Shanghai, China).

### 2.6 Statistical analysis

Results are reported using descriptive statistics. For graph preparation, GraphPad Prism V.8.0 (San Diego, California, USA) was used. PERMANOA analysis was used to determine the statistical significance of sample grouping for β‐diversity. For statistical analysis, SPSS V.15.0 software (Chicago, IL, USA) and R V.4.1.0 were used. Due to the small number of subjects, paired samples Wilcoxon signed ranks test was used to compare values before and after XOS, FOS, and placebo intake. Wilcoxon signed ranks test were used to compare the effect between different groups. Results are expressed as the means ± SD, with *p* value less than .05 considered significant. Multiple Testing using Significance Analysis of Microarrays (SAM). The identification of parameters differentially expressed between groups (unpaired) or within the same group at different time points (paired) and estimation of the False Discovery Rate (FDR) was calculated using the siggenes package in R. Significance was described as FDR ≤ 0.05 and a *q*‐value ≤ .10. Non‐normally distributed data were presented as median (quartiles) and had been analyzed using the Mann–Whitney *U*‐test or Kruskal–Wallis *H* test. Before the statistical analyses, the microbiota data were all performed normalization. Chi‐Square test was used to examine the difference of the proportion of females and males in each group.

## 3 RESULTS

### 3.1 Baseline characteristics of the study population

**FIGURE 1:**
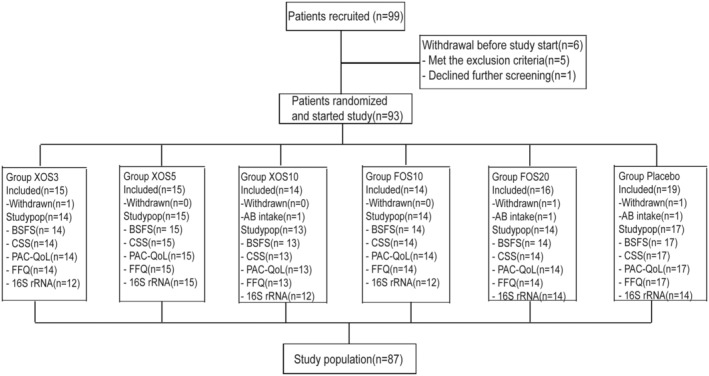
Schematic presentation of the study design. XOS was administered at a dose of 3.0 g/d, 5.0 g/d and 10.0 g/d, FOS was administered at a dose of 10.0 g/d and 20.0 g/d, placebo was administered at a dose of 5.0 g/d.

A total of 99 subjects were screened and randomly assigned to any one of six different groups. Of these, six subjects terminated the study early during the break‐in period; three subjects decided to terminate the study for personal, non‐medical reasons; and three subjects were asked to terminate the study due to use of antibiotics during the break‐in period (Figure 1). Therefore, a total of 87 subjects were included in the study population. 14 were included in the group XOS3, 15 in the group XOS5, 13 in the group XOS10, 14 in the group FOS10, 14 in the group FOS20, and 17 in the group Placebo. Sampling size for this study, all clinical cases with evaluable biospecimens were used during the period of data generation. There was no significant variance between the groups in terms of the proportion of female and male subjects. Specific information about each subject is provided in supplementary information (Table S3).

Baseline demographic and anthropometric characteristics of the six randomly grouped populations are presented in Table 2. No significant differences were observed at baseline among the six randomization groups concerning gender, age, BMI, BSFS, CCCS, and PAC‐QoL scores.

**TABLE 2:** Demographics of the study participants.

Group	Subjects (*n*)	Gender		Age (years)	BMI (kg/m^2^)	BSFS	CCCS	PAC‐QoL
		Female (*n*)	Male (*n*)					
XOS3	14	10	4	56.71 ± 14.01	21.36 ± 1.73	2.35 ± 1.34	13.36 ± 3.15	68.21 ± 20.79
XOS5	15	12	3	55.20 ± 17.55	23.20 ± 3.33	2.60 ± 1.18	12.93 ± 3.33	62.40 ± 19.88
XOS10	13	10	3	45.92 ± 17.40	22.18 ± 2.92	2.62 ± 1.33	11.38 ± 4.91	64.31 ± 15.28
FOS10	14	13	1	46.93 ± 14.66	22.03 ± 2.27	2.43 ± 1.16	12.00 ± 2.60	71.50 ± 15.15
FOS20	14	12	2	51.29 ± 13.82	22.89 ± 2.94	2.29 ± 1.44	13.29 ± 2.97	71.29 ± 11.50
Placebo	17	14	3	53.06 ± 10.76	21.85 ± 2.75	2.35 ± 1.50	13.29 ± 3.31	69.94 ± 22.41

*Note*: XOS3: supplementation with xylo‐oligosaccharides (XOS), 3 g/day, XOS5: supplementation with XOS, 5 g/day. XOS10: supplementation with XOS, 10 g/day, FOS10: supplementation with fructo‐oligosaccharides (FOS), 10 g/day, FOS20: supplementation with FOS, 20 g/day, Placebo: supplementation with placebo 5 g/day.

Abbreviations: BMI, body mass index; BSFS, Bristol Stool Form Scale; CCCS, Cleveland Clinic Constipation Score; PAC‐QoL, Quality of Life Scale for Patients with Constipation.

### 3.2 Gastrointestinal symptoms

**FIGURE 2:**
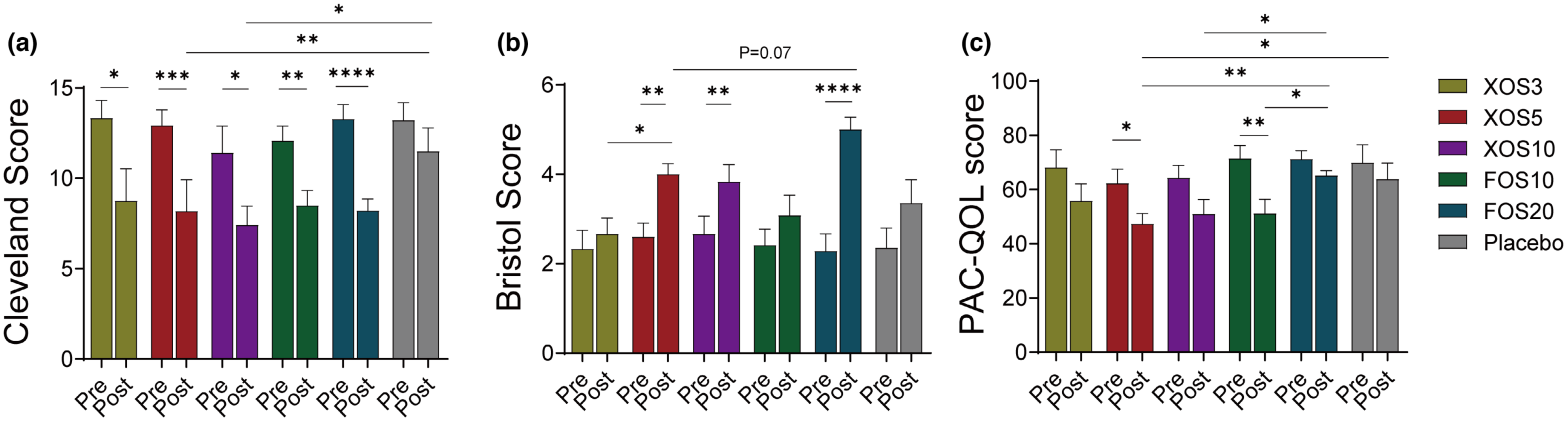
Differences in constipation before and after intervention with different doses of dietary care containing XOS or FOS and placebo. (A) Changes in Cleveland score. (B) Changes in Bristol score. (C) Changes in PAC‐QoL score (“Pre” stands for pre‐intervention and “Post” for post‐intervention).

Regarding gastrointestinal symptoms, Figure 2 demonstrates the changes in relevant questionnaire scores among the six groups of subjects. All intervention groups showed a decline in constipation scores according to the CCCS, with substantial declines in the XOS5, FOS10, and FOS20 constipation scores (Figure 2a). Stool normalization was more pronounced in the XOS5 and XOS10 groups than in the Placebo group in patients with FC (Figure 2b). The stool properties in the patients in the FOS20 group tended to be soft and paste‐like, possibly due to the high dose intake of FOS (Kumar et al., 2020). Patients in FOS10 group exhibited better improvement in gastrointestinal symptoms than did those in FOS20 group after intervention. Patients in XOS5 group showed the highest improvement in symptoms based on Bristol scores, and this improvement was also demonstrated by a significant decrease in CCCS and PAC‐QoL scores (Figure 2c).

### 3.3 Diversity of gut microbiota

The effects of prebiotic interventions on the diversity of the gut microbiota were evaluated by 16S rRNA gene amplicon sequencing. The bacterial diversity information is shown in Appendix S1. We explored the microbial community diversity of these groups based on paired fecal samples. The α‐diversity of the microbiota in subjects in the XOS10 group was unchanged according to the Simpson index. Similarly, the α‐diversity of other groups, based on Chao1 index, Shannon indexes, and Ace index did not change significantly (Figure S1).

**FIGURE 3:**
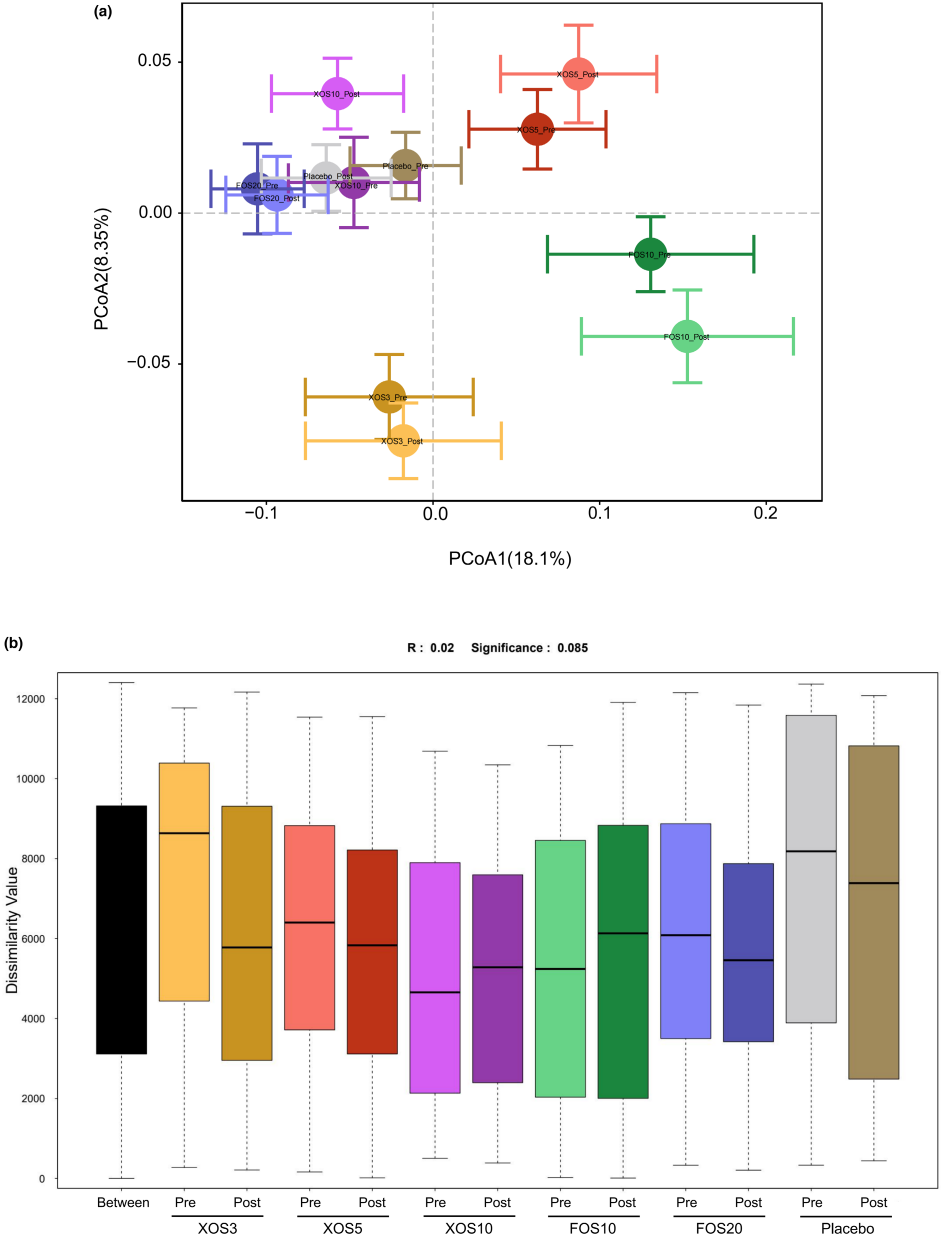
The microbiota diversity of different specimen types.(A) Principal coordinate analysis (PCoA) demonstrating relationships between the microbiota in different specimen types based on a Bray‐Curtis similarity matrix derived from square root transformed OTU‐level data. (B) Unweighted Unifrac ANOSIM analysis between six groups.

We performed principal coordinate analysis (PCoA) on the abundance matrix based on Bray‐Curtis distances (Figure 3a). Before the intervention, there were no significant differences in structures of the gut microbiota among all the groups, (PERMANOVA, *p* > .05). At the end of the intervention, supplementation with FOS and XOS did not result in significant differences in the gut microbiota structure (PERMANOVA, *p* > .05), and there were no significant differences in structure among the six groups (Figure 3b).

### 3.4 Comparison of the abundance of differential gut bacteria

**FIGURE 4:**
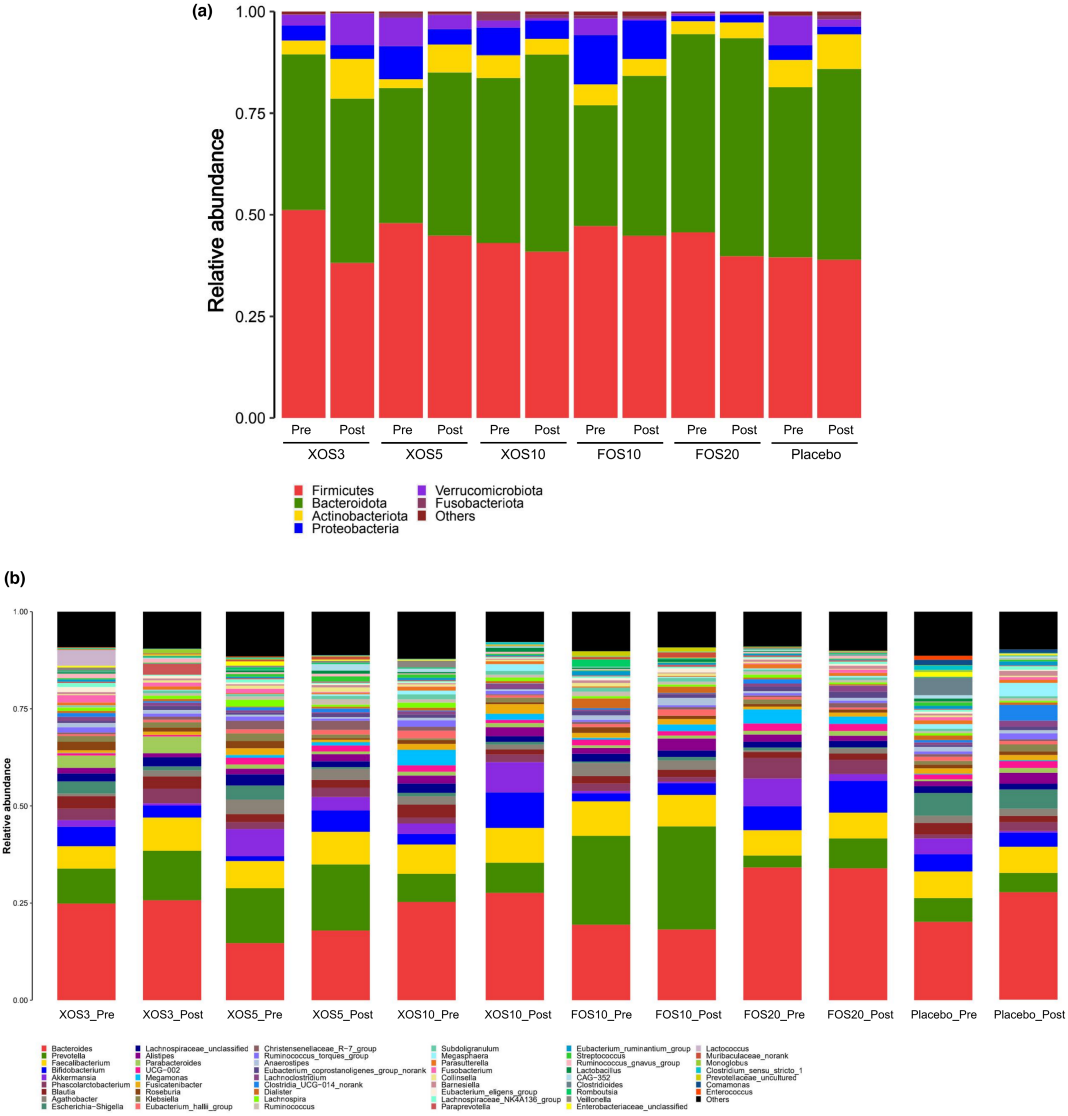
Composition of gut microbiota of human from different groups at phylum (A) and genus level (B).

At the phylum level, *Firmicutes*, *Bacteroidetes*, *Actinobacteriota*, *Proteobacteria*, and *Verrucomicrobiota* were the dominant phylum in the gut microbiota of subjects in all six groups (Figure 4a). At the genus level, the interventions led to the most pronounced changes to *Bifidobacterium* species (Figure 4b). In particular, upward trends in *Bifidobacterium* abundance were observed in the XOS5 and XOS10 groups, but such an upward trend in overall *Bifidobacterium* abundance was not observed in the FOS groups.

### 3.5 Comparison of the microbiota in paired fecal samples

The paired samples Wilcoxon test was performed to compare the operational taxonomic units (OTUs) within each group before and after the intervention (Table 3). The levels of *Bifidobacterium* were significantly enriched by the treatments in the XOS5 (*p* = .008) and XOS10 (*p* = .013) groups, but they were not significantly enriched by the interventions in Placebo, FOS10, or FOS20 groups. *Romboutsia* and *Lachnospiraceae uncultured* (*L. uncultured*) were found to be significantly reduced after treatment in the XOS10 group, and *Collinsella* was found to be significantly reduced after the intervention in the FOS10 group.

**TABLE 3:** Influence of XOS and FOS on the microbiota expressed as feces (Medians and interquartile ranges (IQR)).

	XOS3		XOS5		XOS10		FOS10		FOS20	
	Pre	Post	Pre	Post	Pre	Post	Pre	Post	Pre	Post
*Bifidobacterium*	377.5 (91.5–4022)	513.5 (90–2164)	83.5 (8.25–261.75)	1556.5** (180.25–2753.25)	931 (172–2020)	2361* (1370–5842)	51 (6–569.25)	799 (177–1844)	400.5 (73–5136.5)	394.5 (66.25–4907.25)
*Romboutsia*	18 (5.25–162.75)	28.5 (10.25–108.75)	111.5 (40.5–158)	63 (24–148.75)	49 (10.5–234)	11.5 (10–36.25)*	40.5 (10.5–89.25)	29 (6.75–102)	13 (0–142.5)	31.5 (8–142.75)
*R. gnavus group*	55 (20–559)	53 (2–356.5)	56 (12.25–161.75)	19.5 (12.75–93.25)	51.5 (15–143.5)	28.5 (6.5–125)	15.5 (5.5–40.75)	16.5 (6–38.75)	38.5 (5.25–197)	64* (20.75–292.5)
*L*. uncultured	56.5 (27.75–123.25)	58 (19.5–162)	83.5 (47.75–128.25)	57.5 (34–99.5)	117.5 (95.25–156.25)	62.5* (30.25–106.75)	55.5 (34.25–146.5)	39 (15.25–90.75)	47 (5.5–123.5)	74 (40.75–147.25)
*Collinsella*	53 (0–183.5)	23.5 (0–236.25)	115.5 (42–400.5)	192.5 (47.5–482.75)	142 (56.25–241)	123.5 (73–160.25)	157 (12.25–350.5)	64.5* (7.25–236)	60.5 (0–200.75)	34 (0–144.25)
*Escherichia‐Shigella*	426 (19.25–2277.5)	103 (18.5–466.5)	67.5 (38.25–443)	41.5 (11.75–106.5)	129.5 (34.5–610)	141 (37.75–225.25)	36.5 (14.25–283)	94* (17–402.5)	87.5 (27.5–402.25)	12.5 (2.5–136.75)
*Roseburia*	419 (47.5–1371.5)	110 (27.25–735.75)	387.5 (161.25–844.25)	84** (25.75–280.25)	277 (35.75–490.75)	114.5 (18.75–250.25)	144.5 (61.75–1349.25)	264 (27.75–436.25)	54 (2.5–780.25)	76.5 (15–350.25)

*Note*: Paired samples Wilcoxon signed ranks test, *p*‐value relates to comparison of Pre and Post. *p_adjust_ < 0.05, **p_adjust_ < 0.01, ***p_adjust_ < 0.001. “Pre” pre‐intervention, “Post” for post‐intervention. *R. gnavus* group (*Ruminococcus_gnavus_group*), *L. uncultured* (*Lachnospiraceae uncultured*).

Abbreviations: FOS10, supplementation with fructo‐oligosaccharides (FOS), 10 g/d; FOS20, supplementation with FOS, 20 g/d; Placebo, supplementation with placebo, 5 g/d; XOS3, supplementation with xylo‐oligosaccharides (XOS), 3 g/d; XOS5, supplementation with XOS, 5 g/d; XOS10, supplementation with XOS, 10 g/d. Corrected <10/01/2024>; In the originally published version, columns FOS10 and FOS20 in Table 3 contained erroneously duplicated data. The table has been updated with the correct data.

Interestingly, we found that the abundance of *Roseburia* was significantly decreased after the intervention in the XOS5 group (*p* < .01). *Roseburia* species have been shown to efficiently utilize β‐mannan to produce a large amount of butyric acid, which can then exert a variety of probiotic functions in the intestinal environment (La Rosa et al., 2019). Conversely, *Ruminococcus*_gnavus_group (*R*. gnavus group) may play a negative role in digestive function, as they synthesize and secrete glucorhamnan, which induces dendritic cells to secrete tumor necrosis factor, leading to inflammation and mucosal oxidative stress (Henke et al., 2019). The abundance of *R. gnavus group* in the feces of FC patients increased in the FOS20 group. In addition, the abundance of *Escherichia‐Shigella*, which represent some of the main pathogens causing intestinal infections, significantly increased in the FOS10 group (*p* < .05) and tended to increase in the XOS10 group (*p* > .05).

### 3.6 Comparison of fecal samples microbiota increments among groups

We used the Kruskal‐Wallis test to compare microbiota abundance increments for independent samples from the six intervention groups. The results showed that the increments of *Bifidobacterium*, *Eubacterium*_ventriosum_group, *L. uncultured*, and *Ruminococcus*_torques_group were significantly different relative to that in the Placebo group (Table 4).

**TABLE 4:** Influence of XOS, FOS, and placebo on the microbiota abundance increment expressed as feces (Medians and interquartile ranges (IQR)).

	XOS3 median (IQR)	XOS5 median (IQR)	XOS10 median (IQR)	FOS10 median (IQR)	FOS20 median (IQR)	Placebo median (IQR)
Δ *Bifidobacterium*	−27.5 (−2836 to 486.5)	1384 (55.75 to 2544)**	1113 (24.5 to 4804)*	399.5 (22 to 1279)	84 (−1428 to 3051)	5.5 (−304.5 to 131.5)
Δ *Eubacterium*_ventriosum_group	7.5 (0 to 69.5)**	70.5 (1 to 130.8)***	0.5 (−11 to 30.5)*	2 (−112.3 to 57.5)	6 (−69.25 to 58.5)*	−27.5 (−173 to 0)
Δ *Ruminococcus*_torques_group	−75.5 (−319 to 24.5)	−95 (−640.8 to 32)	−333 (−574.5 to −92)*	−4 (−106.5 to 63.5)	66.5 (−117.5 to 268)	−1 (−93 to 279.8)
Δ *Lachnospiraceae uncultured*	10 (−27 to 61.5)	−23 (−83 to 32.75)	−62 (−122.3 to −14)*	−30 (−70.75 to 2.25)	18 (−33 to 76.5)	0.5 (−41.75 to 10)

*Note*: Δ Increment of the abundance of the gut bacteria. Wilcoxon test, *p* value is compared to placebo, *p_adjust_ <.05, **p_adjust_ <.01, ***p_adjust_ <.001. XOS3: supplementation with xylo‐oligosaccharides (XOS), 3 g/day; XOS5: supplementation with XOS, 5 g/day; XOS10: supplementation with XOS, 10 g/day; FOS10: supplementation with fructo‐oligosaccharides (FOS), 10 g/day; FOS20: supplementation with FOS, 20 g/day; Placebo: supplementation with placebo, 5 g/day.

The abundance of *Bifidobacterium* increased to a significantly greater extent in the XOS5 (*p* < .01) and XOS10 (*p* = .017) groups relative to the Placebo group. The abundance of *Eubacterium_ventriosum_group* not only increased significantly over the course of the intervention in the XOS5 group (Table 3), but this change was also greater than that observed in the Placebo group during the 1‐month intervention (Table 4). The abundance of *Ruminococcus*_torques_group and *L. uncultured* underwent a greater reduction in the XOS10 group than in the Placebo group.

## 4 DISCUSSION

In this double‐blind, randomized, placebo‐controlled 1‐month study, we found that administration of a dietary supplement containing XOS at a dose of 5 g/day showed a better effect on FC patients as compared to similar supplementation with FOS at doses of 10 or 20 g/day, and no side effects were observed in subjects in the XOS5 group. While major changes in flora structure were not detected, an obvious enrichment of *Bifidobacterium* was observed in the XOS5 group following the 1‐month intervention. This finding indicates that XOS dietary care may be more effective as an FC intervention than FOS.

The results of follow‐up visits showed most subjects in the study experienced some level of relief from constipation symptoms over the course of the intervention. Patients' self‐ratings of other gastrointestinal symptoms, as assessed by the CCCS, were more strongly improved when treated with XOS as compared to treatment with Placebo. In addition, a dose of 5 g/day XOS was determined to be optimal, as subjects treated with 5 g/day XOS experienced better relief in gastrointestinal symptoms than did those who received 3 or 10 g/day XOS.

FOS at a dose of 10 g/day significantly reduced CCCS and PAC‐QoL scores, but it resulted in non‐significant changes to stool properties, as shown by increased Bristol scores (3.14 ± 1.46 after the intervention). Bristol scores are considered to be more objective than CCCS and PAC‐QoL scores, which are mainly based on patient perceptions. The general tolerance of patients with constipation for the symptoms associated with constipation may be an explanation for the disparity in the results of CCCS and PAC‐QoL scoring assessment relative to the Bristol score assessment.

In terms of flatulence, mild to moderate flatulence is often caused by gas production during the fermentation of prebiotic compounds, such as inulin and FOS (Bouhnik et al., 1999). When we explored the changes in constipation symptoms in patients, we found that the degree of flatulence tended to become reduced in each intervention group over the course of the study. Compared to placebo, the gastrointestinal function in FC patients was more strongly improved after interventions with 5 or 10 g/day XOS or with 10 g/day FOS. In contrast, some patients in the FOS20 group experienced increased flatulence, diarrhea, and decreased appetite. It was also evident that the patients in the FOS20 group tended to have a decreased intake of staple foods based on the FFQ data (Table S1).

XOS has been shown to stimulate the proliferation of *Bifidobacterium*, increase the content of short‐chain fatty acids (SCFAs) in the host, and inhibit pathogenic bacteria by promoting the innate immune response (Olszak et al., 2012), all of which are beneficial for gastrointestinal health. SCFAs, the primary metabolites produced by bacterial fermentation of dietary fiber in the gastrointestinal tract, have regulatory roles in lipid and glucose metabolism, in anti‐inflammatory and immune responses, and in the maintenance of gut barrier integrity (Nogal et al., 2021). Additionally, SCFAs play a crucial role in crosstalk along the gut‐microbiota‐brain axis (Dalile et al., 2019; Silva et al., 2020). In animal models, the addition of 7%–10% of XOS has been shown to be associated with no observable side effects (Gobinath et al., 2010). XOS supplementation has also been shown to be effective and safe in human subjects. For example, Tateyama et al. (2005) found that oral supplementation of XOS relieved severe constipation symptoms in pregnant women with FC, and this XOS dietary care was associated with no adverse effects. Notably, the current Chinese National Health and Wellness Commission New Resource Food Catalogue Announcement No. 20, 2014 (http://www.nhc.gov.cn) stipulates that the daily human intake of XOS should not exceed 3 g. However, in this study, a daily intake of 3 g XOS was less effective at relieving FC symptoms than was a daily intake of 5 g, and this larger dose did not lead to side effects.

Previous studies have shown that prebiotic supplementation can alter the abundance of gut microbes, especially regarding *Bifidobacterium*. Okazaki et al. (1990) discovered that XOS observed that supplementation with XOS increased the relative abundance of *Bifidobacterium* within the total intestinal microbiota; in that study, the abundance of *Bifidobacterium* decreased after discontinuation of XOS. FOS can also lead to the proliferation of *Bifidobacterium* (Souza et al., 2018), at higher doses than used for XOS supplementation or in combined treatments with other prebiotic agents (Bomhof et al., 2019; Hedin et al., 2021). In the present study, α‐diversity changes in gut microbiota based on the Shannon, Simpson, Chao, and Ace index were not observed during the intervention period, except in the XOS10 group. In addition, upon analyzing the β‐diversity of the gut microbiota, we found that XOS and FOS did not significantly alter the structure of the gut microbiota compared to placebo.

Therefore, we conclude that XOS led to a targeted enrichment of *Bifidobacterium* without affecting the overall structures of the gut microbiota when employed at a low dosage. Overall, XOS may help stimulate intestinal motility by regulating *Bifidobacterium*, and thus promoting excretion.

Inhibition of *Ruminococcus*_torques_group within the intestinal microbiome is known to be beneficial for the control of body fat levels. A reduction in *Ruminococcus*_torques_group was reported to be involved in mediating the beneficial effects of prebiotics in non‐obese diabetic mice (Hnninen et al., 2017). *Eubacterium*_ventriosum_group can convert polysaccharides into SCFAs, increasing the efficiency of energy collection (Kasai et al., 2015). Compared to the Placebo group, the abundance of *Ruminococcus*_torques_group decreased most significantly in the XOS10 group (*p* < .05), while *Eubacterium*_ventriosum_group increased dramatically in the XOS5 group (*p* < .001) (Table 4). *L. uncultured* has been reported to be a key microbial marker that can differentiate the gut microbiota of non‐diarrheic and diarrheic (Ma et al., 2020). In the present study, *L. uncultured* was found to be reduced in the XOS10 group at the end of the intervention as compared to levels in the same subjects before the intervention (Table 3), and the levels after the intervention were significantly lower than those in the Placebo group (Table 4). *R. gnavus group* synthesizes and secretes glucorhamnan, which induces dendritic cells to secrete tumor necrosis factor, leading to inflammation and mucosal oxidative stress (Henke et al., 2019), meaning that the abundance of this type of bacterium is likely to be associated with poor digestive tract function. The abundance of *R. gnavus group* in the feces of FC patients increased in the FOS20 group may have led to increased intestinal inflammation in this group, potentially resulting in diarrhea, flatulence and other symptoms. Additionally, we cannot exclude the possibility that large dosages of FOS (20 g/day) caused diarrhea by increasing stool osmolality and water absorption (Tian et al., 2023). *Escherichia‐Shigella*, one of the main pathogens causing intestinal infections, significantly increased in the FOS10 group (*p* < .05) and tended to increase in the XOS10 group (*p* > .05). On the other hand, a genus of bacteria with a potentially beneficial impact on digestive health is *Roseburia*, which can efficiently utilize β‐mannan to produce a large amount of butyric acid, which can exert a variety of probiotic functions in the intestinal environment. The abundance of *Roseburia* decreased after the 5 g XOS intervention (*p* < .01). In general, we found that at the genus level, XOS intervention reduced multiple types of butyrate‐producing bacteria, including *Roseburia* (Kumar et al., 2020) and *Romboutsia* (Chen et al., 2021). It was inferred that *Bifidobacterium* proliferated and produced large amounts of lactic acid, which inhibited the growth of conditionally pathogenic bacteria, while hindering the growth of some butyric acid‐producing bacteria.

In addition to the use of prebiotic agents as treatments or supplements, the addition of prebiotics to foods has also been a widely accepted practice (Precup et al., 2022). The modification of foods to enhance the microbiome was first reported as the concept of Microbiota‐Directed Food (MDF) in 2017 (Hibberd et al., 2017), and it specifically referred to classes of foods that can improve the structure of gut microbiota in a targeted way. This effort can result in the precise and personalized regulation of the flora and thus serve as an innovative approach to assist in the treatment of disease. In the present study, care‐givers tracked and monitored FC patients' diet, and their gastrointestinal symptoms. Importantly, the relationship of the caregivers with the patients provided them insight into their psychological status, as it has been well established that patients with long‐term gastrointestinal symptoms are more likely to experience psychological problems such as anxiety and depression (Simpson et al., 2021). These psychological problems can in turn have a deleterious impact on gastrointestinal symptoms, potentially via the gut‐brain axis. Therefore, the study of targeted regulation of gut microbiota and multi‐sectoral cooperation are critical for the future development of precision nutrition.

In the current study, the majority of the subjects were adults who were more than 40 years old, this age distribution is consistent with the fact that age is an important risk factor for the development of FC (Arco et al., 2022; Barberio et al., 2021). We also found that female subjects were more motivated than male subjects during the recruitment period. This difference might be explained by the idea that females tend to have higher levels of anxiety and depression, and females have been found to be more frequently aware of the symptoms (Cheng et al., 2003; Zhang et al., 2022). For example, a cross‐sectional questionnaire‐based survey among Taiwanese adolescents (12–18 years old) found that female students were likely to rate their health status as being poorer than males (Tsai et al., 2014). In China, gender difference was strongly related with self‐rated health across the sample (Jia et al., 2014), and in another study, females were more likely to act on symptoms and to adopt a sick persona (Cheng et al., 2015). All of these gender differences result in the high motivation regarding seeking strategies in females.

We acknowledge several limitations in our study. The size of this study was relatively small. In addition, we cannot exclude the possibility that the increased level of prebiotic agents influenced constipation symptoms by increasing the osmolality of the stool and thus drawing additional water into the stool. In terms of the reporting of behaviors by the study subjects, we note that while FFQ can capture individual‐level dietary patterns, they are also prone to recall bias.

## 5 CONCLUSION

Supplementation with XOS can cause a targeted enriching of *Bifidobacterium* in the gut microbiota and can lead to improvements to constipation symptoms at lower dosages than can FOS supplementation. In addition, we found that interventions using XOS were not associated with side effects such as diarrhea and flatulence. This study thus provides a new strategy of nutrition management for patients with FC. In addition, we propose that the prevention of constipation and the improving of gut microbiota could be easily performed through the targeted consumption of foods with XOS.

## AUTHOR CONTRIBUTIONS

**Wanya Yi:** Conceptualization (equal); data curation (equal); formal analysis (equal); investigation (equal); writing – original draft (lead). **Qinyue Wang:** Data curation (equal); formal analysis (equal); investigation (equal); writing – original draft (equal). **Yuzheng Xue:** Data curation (equal); formal analysis (equal); investigation (equal); writing – original draft (equal). **Hong Cao:** Investigation (equal); project administration (equal). **Ruijuan Zhuang:** Data curation (equal); writing – review and editing (equal). **Dan Li:** Funding acquisition (equal); investigation (equal). **Jiai Yan:** Data curation (equal); investigation (equal). **Ju Yang:** Data curation (equal); investigation (equal). **Yanping Xia:** Project administration (equal); resources (equal); supervision (equal). **Feng Zhang:** Conceptualization (equal); funding acquisition (equal); project administration (equal); resources (equal); writing – review and editing (equal).

## FUNDING INFORMATION

This work was supported by National Natural Science Foundation of China (81870544, 81870594); the Natural Science Foundation of Jiangsu Province (BK20181132); Scientific Research Project of Jiangsu Commission of Health (M2021055); the funding for Leading Talents and Advanced Talents in Medical and Health Profession in Wuxi Taihu Lake Talent Plan; Science and Technology Program Project of Jiangsu Market Supervision and Administration (KJ2022028); Jiangsu Scientific Research Project of Elderly Health (LK2021035); Jiangsu Scientific Research Project of Women's and Children's Health (F201741); Scientific Research Project of Wuxi Commission of Health (ZZ003, Q201762); Wuxi Scientific and Technological Development Project (N20192024, N20191001, Y20212001); Translational Medicine Research Program of Wuxi Translational Medicine Center (2020ZHYB08).

## CONFLICT OF INTEREST STATEMENT

All authors certify that they have no affiliations with or involvement in any organization or entity with any financial interest or non‐financial interest in the subject matter or materials discussed in this manuscript.

## ETHICS STATEMENT

Approval of the Human Subjects Committee of the Affiliated Hospital of Jiangnan University was obtained before the beginning of the study (Number: IEC201803001), and was registered at the Chinese Clinical Trial Registry (ChiCTR1800015888).

## INFORMED CONSENT

Written informed consent was obtained from all study participants.

## Supporting information


Appendix S1

## Data Availability

The data that support the findings of this study are available on request from the corresponding author. The data are not publicly available due to privacy or ethical restrictions.
