# Supplementary material for: Xylo‐oligosaccharides improve functional constipation by targeted enrichment of Bifidobacterium
Source: Food Sci Nutr. 2023 Nov 27;12(2):1119–32. doi: 10.1002/fsn3.3827 (PMC10867466; doi:10.1002/fsn3.3827)
Supplement: Supplementary file 1 — Appendix S1 [file FSN3-12-1119-s001.docx]

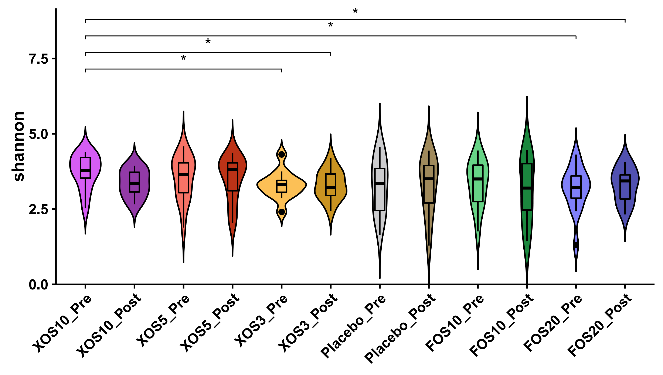

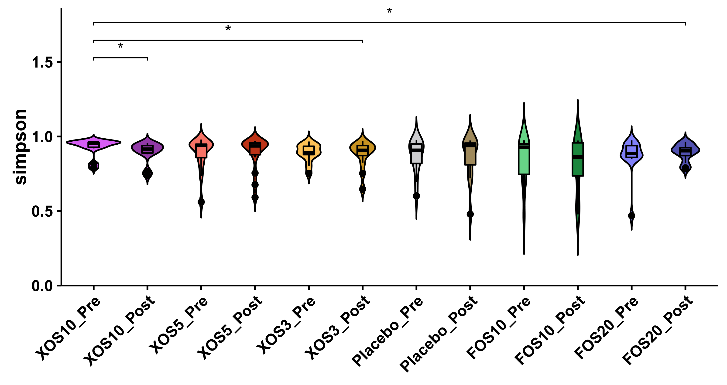

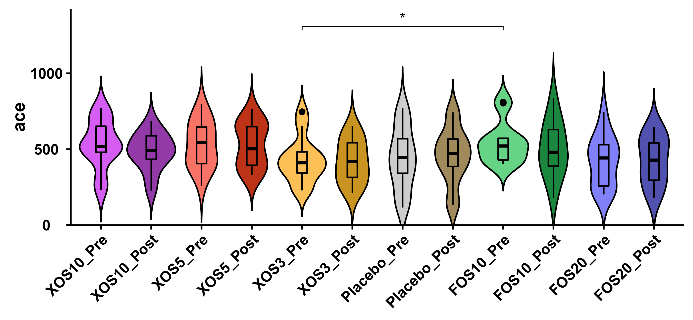

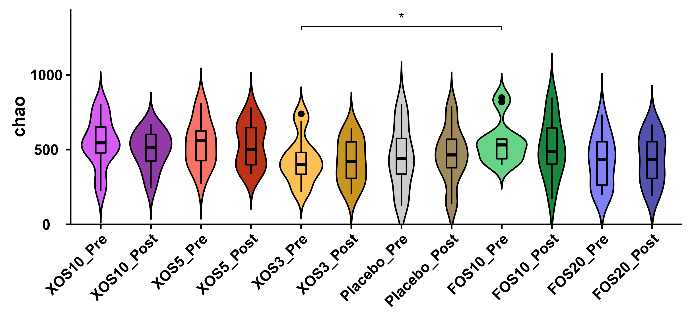


**Figure S1** Comparison of α diversity indexes (Shannon, Simpson, ACE and Chao1) between different groups.

Table S1. Daily dietary intake of each group (XOS, FOS and placebo)

| Food groups | XOS3 | XOS5 | XOS10 | FOS10 | FOS20 | Placebo |
| --- | --- | --- | --- | --- | --- | --- |
| Rice  Porridge and rice in soup  Noodles, steamed bun and bread  Wonton, dumplings  Whole grains  Tubers  Dark Green Vegetables  Light vegetables  Fruit  Mushrooms  Red meat  Poultry and organs  Processed and cooked meat  Fish and shrimp  Eggs (Duck eggs, chicken eggs)  Seafood  Milk  Soya bean and its products  Fried foods  Cakes and desserts  Nuts  Drinks  Beer  Alcoholic beverages (Chinese Baijiu) | 73.34(10.00,150.00)  46.68(10.83,98.33)  33.34(10.00,89.99)  13.33(6.67,66.67)  11.66(4.17,29.17)  18.34(6.68,86.67)  31.68(13.33,49.17)  38.34(24.16,49.17)  73.33(29.18,100.00)  6.68(3.33,19.99)  30.00 (10.00,49.17)  30.00 (11.68,45.01)  5.84(1.67,24.17)  8.34(1.66,23.34)  25.84(19.58,46.67)  3.34(0.00,6.67)  146.70(10.00,215.00)  5.84(3.33,21.68)  5.00 (0.00,89.99)  9.99(3.35,67.50)  5.00(2.08,26.68)  5.00(0.00,59.17)  0.00(0.00,8.13)  0.00(0.00,3.83) | 146.70(46.68,200.00)  40.00(26.68,80.00)  50.00(30.00,110.00)  26.68(6.68,30.00)  13.33(6.68,50.00)  10.00 (3.34,46.68)  46.68(23.33,100.00)  26.68(10.00,36.68)  46.68(23.33,73.33)  10.00(3.33,23.33)  46.68(13.33,80.00)  10.00 (6.68,40.00)  5.00 (3.33,6.68)  10.00(3.33,26.68)  11.67(5.00,36.68)  5.00(1.67,6.68)  60.00 (10.00,140.00)  6.68(3.33,36.68)  6.68(1.67.13.33)  6.68(5.00,26.68)  3.33(3.33,10.00)  6.67(0.00,23.33)  0.00(0.00,21.67)  0.00(0.00,0.00) | 70.00(30.00,200.00)  28.34(6.68,72.50)  58.34(4.17,102.50)  10.00(6.68,37.5)  6.66(3.33,25.83)  18.33(10.00,130.8)  61.66(19.17,107.50)  41.68(8.75,72.50)  36.68(14.17,73.33)  6.67(3.33,22.51)  26.68(13.33,66.66)  33.34(6.67,72.50)  8.33(2.08,10.00)  11.66(4.16,40.00)  24.16(11.67,49.17)  5.00(0.42,6.67)  13.34(4.17,90.00)  5.01(1.65,28.33)  13.33(2.92,30.00)  13.33(3.33,30.00)  2.50(0.00,10.00)  6.67(0.00,23.33)  0.00(0.00,13.54)  0.00(0.00,8.75) | 100.00(17.50,150.00)  25.00 (6.68,72.50)  40.00 (14.17,73.33)  25.00 (7.51,137.50)  6.68(3.74,67.50)  10.00 (2.08,23.34)  46.68(36.68,82.49)  41.68(24.16,50.00)  48.34(26.68,100.00)  3.33(3.33,6.68)  46.68(31.67,67.49)  28.34(7.08,82.49)  3.33(0.42,12.49)  6.68(3.75,21.68)  24.16(5.42,36.68)  3.33(1.67,9.17)  83.33(5.83,186.7)  2.50(1.65,10.00)  10.00 (3.33,17.08)  6.68(3.75,25.00)  6.67(2.08,33.34)  9.17(0.00,22.50)  0.00(0.00,0.00)  0.00(0.00,0.00) | 18.34(6.67,236.70)  35.00 (14.17,89.99)  86.66(12.51,100.00)  8.34(6.68,37.50)  11.66(3.33,26.68)  10.00 (7.51,71.67)  50.00(10.83,95.00)  28.34(23.33,71.67)  61.66(15.00,88.33)  3.33(3.33,9.17)  36.68(10.00,89.99)  10.00 (6.67,47.50)  5.84(0.83,15.83)  5.00(2.08,10.00) *  28.34(1.25,73.33)  3.33(0.00,21.67)  60.00(25.00,145.00)  9.17(4.16,19.99)  20.00 (3.33,107.5)  16.67(3.33,70.00)  8.33(3.33,26.68)  7.50(0.00,140.0)  18.06(0.00,59.58)  0.00(0.00,0.00) | 20.00(6.68,125.00)  13.33(6.68,57.50)  76.66 (41.68,119.20)  35.00 (8.34,83.33)  10.00 (3.34,30.00)  16.66(6.26,100.00)  31.68(19.99,100.00)  38.34(10.00,78.33)  48.34(25.00,100.00)  6.68(3.33,10.00)  38.34(25.00,94.99)  20.00 (10.00,55.01)  8.34(2.91,23.33)  28.34(9.17,30.00)  25(10.00,29.18)  5.83(3.33,30.00)  93.33(18.33,200.00)  8.34(1.65,15.83)  5.34(5.00,29.99)  11.66(7.92,30.01)  5.01(2.91,16.67)  10.00(0.00,20.83)  0.00(0.00,54.17)  0.00(0.00,0.00) |

Data were presented as median (quartiles), * Kruskal-Wallis H test, P value is compared to placebo，**P*<0.05, ***P*<0.01, *** *P*<0.001.

XOS3: supplementation with xylo-oligosaccharides (XOS), 3 g/d, XOS5: supplementation with XOS, 5 g/d; XOS10: supplementation with XOS, 10 g/d, FOS10: supplementation with fructo-oligosaccharides (FOS), 10 g/d, FOS20: supplementation with FOS, 20 g/d, Placebo: supplementation with placebo 5 g/d.

Table S2. Score of CCCS in pre-experiment (XOS, FOS and placebo)

| Group | Subjects  (n) | Age  (years) | BMI  (Kg/m2) | CCCS  pre | CCCS  post |
| --- | --- | --- | --- | --- | --- |
| XOS3 | 5 | 55.20±13.70 | 22.94±0.81 | 13.80±2.17 | 9.00±3.81* |
| XOS5 | 5 | 58.20±9.31 | 23.91±4.44 | 13.80±0.84 | 5.80±2.49*** |
| XOS10 | 5 | 46.40±21.23 | 22.94±3.39 | 14.00±5.43 | 7.00±2.74* |
| FOS10 | 5 | 48.80±17.80 | 22.33±3.09 | 11.60±3.51 | 8.40±1.67 |
| FOS20 | 5 | 42.80±13.52 | 22.57±1.44 | 13.80±4.32 | 7.60±1.52* |
| Placebo | 5 | 50.20±10.62 | 21.94±3.17 | 13.00±4.58 | 11.00±4.00 |

XOS3: supplementation with xylo-oligosaccharides (XOS), 3 g/d, XOS5: supplementation with XOS, 5 g/d; XOS10: supplementation with XOS, 10 g/d, FOS10: supplementation with fructo-oligosaccharides (FOS), 10 g/d, FOS20: supplementation with FOS, 20 g/d, Placebo: supplementation with placebo 5 g/d. BMI: body mass index. CCCS: Cleveland Clinic Constipation Score. Paired samples t test. **P*<0.05, ***P*<0.01, *** *P*<0.001.*P*-value is the comparison of Pre and Post

Table S3. Specific information about each subject.

| Group | Number | Gender  (F/M) | Age  (years) | BMI  (kg/m^2^) | BSFS | CCCS | PAC-QoL |
| --- | --- | --- | --- | --- | --- | --- | --- |
| XOS3 | XOCC00101 | F | 44 | 21.94 | 4 | 14 | 59 |
|  | XOCC00301 | M | 62 | 19.72 | 3 | 15 | 95 |
|  | XOCC00401 | M | 33 | 19.38 | 1 | 12 | 69 |
|  | XOCC00501 | M | 63 | 20.52 | 3 | 8 | 37 |
|  | XOCC00601 | F | 38 | 23.23 | 1 | 13 | 63 |
|  | XOCC00701 | F | 65 | 23.59 | 4 | 17 | 112 |
|  | XOCC00801 | F | 33 | 21.48 | 1 | 15 | 80 |
|  | XOCC00901 | F | 75 | 21.34 | 4 | 16 | 44 |
|  | XOCC01001 | F | 55 | 18.49 | 4 | 7 | 58 |
|  | XOCC01101 | F | 59 | 22.22 | 1 | 14 | 85 |
|  | XOCC01201 | F | 67 | 23.42 | 1 | 18 | 41 |
|  | XOCC01301 | F | 70 | 23.71 | 1 | 11 | 75 |
|  | XOCC01401 | M | 68 | 20.31 | 2 | 12 | 68 |
|  | XOCC01501 | F | 62 | 19.71 | 3 | 15 | 69 |
| XOS5 | XOCB00101 | F | 53 | 20.62 | 2 | 21 | 98 |
|  | XOCB00201 | F | 65 | 24.89 | 3 | 14 | 14 |
|  | XOCB00301 | F | 63 | 16.94 | 2 | 14 | 64 |
|  | XOCB00401 | M | 61 | 26.23 | 3 | 8 | 71 |
|  | XOCB00501 | F | 71 | 21.48 | 1 | 15 | 44 |
|  | XOCB00601 | M | 65 | 18.95 | 2 | 14 | 68 |
|  | XOCB00701 | F | 54 | 22.10 | 3 | 13 | 59 |
|  | XOCB00801 | F | 74 | 29.67 | 1 | 14 | 91 |
|  | XOCB00901 | F | 70 | 27.34 | 3 | 13 | 59 |
|  | XOCB01001 | F | 59 | 20.96 | 4 | 11 | 68 |
|  | XOCB01101 | F | 73 | 24.14 | 4 | 17 | 70 |
|  | XOCB01201 | F | 41 | 24.46 | 5 | 9 | 61 |
|  | XOCB01301 | M | 18 | 25.76 | 2 | 10 | 51 |
|  | XOCB01401 | F | 36 | 21.88 | 3 | 11 | 73 |
|  | XOCB01501 | F | 25 | 22.60 | 1 | 10 | 45 |
| XOS10 | XOCA00101 | F | 47 | 22.22 | 2 | 4 | 58 |
|  | XOCA00201 | F | 50 | 22.66 | 2 | 13 | 85 |
|  | XOCA00401 | F | 20 | 16.98 | 4 | 13 | 75 |
|  | XOCA00501 | F | 26 | 21.51 | 4 | 16 | 60 |
|  | XOCA00601 | M | 36 | 24.84 | 1 | 21 | 87 |
|  | XOCA00701 | F | 64 | 27.34 | 3 | 14 | 67 |
|  | XOCA00801 | F | 32 | 18.37 | 5 | 6 | 34 |
|  | XOCA00901 | M | 71 | 24.91 | 2 | 15 | 75 |
|  | XOCA01001 | F | 68 | 25.01 | 1 | 7 | 68 |
|  | XOCA01101 | F | 27 | 20.39 | 4 | 6 | 40 |
|  | XOCA01201 | F | 50 | 19.72 | 1 | 8 | 58 |
|  | XOCA01301 | F | 40 | 21.34 | 3 | 14 | 65 |
|  | XOCA01401 | M | 66 | 22.99 | 2 | 11 | 64 |
| FOS10 | XOCE00101 | F | 69 | 21.88 | 4 | 16 | 103 |
|  | XOCE00201 | M | 34 | 25.07 | 3 | 12 | 68 |
|  | XOCE00301 | F | 50 | 27.27 | 3 | 11 | 71 |
|  | XOCE00401 | F | 38 | 20.32 | 1 | 7 | 52 |
|  | XOCE00501 | F | 62 | 20.03 | 1 | 10 | 58 |
|  | XOCE00601 | F | 25 | 19.53 | 1 | 14 | 66 |
|  | XOCE00701 | F | 31 | 21.88 | 4 | 13 | 74 |
|  | XOCE00801 | F | 38 | 19.65 | 4 | 12 | 80 |
|  | XOCE00901 | F | 32 | 23.63 | 2 | 8 | 96 |
|  | XOCE01001 | F | 56 | 22.43 | 1 | 16 | 45 |
|  | XOCE01101 | F | 42 | 23.01 | 3 | 14 | 71 |
|  | XOCE01201 | F | 50 | 23.15 | 2 | 12 | 74 |
|  | XOCE01301 | F | 68 | 19.81 | 2 | 11 | 71 |
|  | XOCE01401 | F | 62 | 20.81 | 3 | 12 | 72 |
| FOS20 | XOCF00101 | F | 21 | 21.88 | 1 | 15 | 70 |
|  | XOCF00201 | F | 59 | 23.88 | 3 | 8 | 53 |
|  | XOCF00301 | F | 34 | 23.81 | 1 | 13 | 64 |
|  | XOCF00501 | F | 62 | 19.53 | 2 | 13 | 65 |
|  | XOCF00601 | F | 38 | 22.39 | 6 | 11 | 83 |
|  | XOCF00701 | F | 54 | 24.65 | 3 | 15 | 80 |
|  | XOCF00801 | F | 34 | 20.44 | 2 | 13 | 66 |
|  | XOCF00901 | F | 56 | 22.86 | 1 | 20 | 96 |
|  | XOCF01001 | F | 59 | 26.84 | 1 | 11 | 55 |
|  | XOCF01201 | F | 55 | 22.10 | 2 | 15 | 72 |
|  | XOCF01301 | F | 58 | 29.67 | 1 | 12 | 74 |
|  | XOCF01401 | M | 59 | 23.57 | 3 | 17 | 66 |
|  | XOCF01501 | F | 70 | 20.58 | 2 | 12 | 83 |
|  | XOCF01601 | M | 59 | 18.34 | 4 | 11 | 71 |
| Placebo | XOCD00101 | F | 37 | 23.53 | 3 | 13 | 87 |
|  | XOCD00201 | M | 58 | 25.61 | 2 | 11 | 41 |
|  | XOCD00301 | F | 56 | 19.03 | 1 | 6 | 23 |
|  | XOCD00401 | M | 56 | 20.20 | 1 | 12 | 90 |
|  | XOCD00501 | F | 64 | 19.53 | 3 | 18 | 101 |
|  | XOCD00601 | F | 41 | 20.96 | 1 | 16 | 66 |
|  | XOCD00701 | F | 51 | 26.67 | 2 | 13 | 57 |
|  | XOCD00801 | F | 41 | 20.08 | 1 | 17 | 70 |
|  | XOCD00901 | F | 39 | 19.10 | 2 | 13 | 88 |
|  | XOCD01001 | F | 69 | 22.48 | 4 | 9 | 46 |
|  | XOCD01101 | F | 43 | 20.93 | 6 | 12 | 83 |
|  | XOCD01301 | F | 70 | 27.39 | 1 | 12 | 56 |
|  | XOCD01401 | F | 56 | 24.35 | 5 | 13 | 59 |
|  | XOCD01501 | F | 69 | 22.22 | 1 | 20 | 112 |
|  | XOCD01601 | M | 50 | 20.90 | 2 | 12 | 69 |
|  | XOCD01701 | F | 54 | 18.90 | 2 | 14 | 70 |
|  | XOCD01901 | F | 48 | 19.53 | 2 | 15 | 71 |

XOS3: supplementation with xylo-oligosaccharides (XOS), 3 g/d, XOS5: supplementation with XOS, 5 g/d; XOS10: supplementation with XOS, 10 g/d, FOS10: supplementation with fructo-oligosaccharides (FOS), 10 g/d, FOS20: supplementation with FOS, 20 g/d, Placebo: supplementation with placebo 5 g/d. Gender(F/M): Females/Males. BMI: body mass index. BSFS: Bristol Stool Form Scale, CCCS: Cleveland Clinic Constipation Score, PAC-QoL: Quality of Life Scale for Patients with Constipation.
